# Blood pressure monitoring during anesthesia induction using PPG morphology features and machine learning

**DOI:** 10.1371/journal.pone.0279419

**Published:** 2023-02-03

**Authors:** Clémentine Aguet, João Jorge, Jérôme Van Zaen, Martin Proença, Guillaume Bonnier, Pascal Frossard, Mathieu Lemay

**Affiliations:** 1 Signal Processing Group, Swiss Center for Electronics and Microtechnology (CSEM), Neuchâtel, Switzerland; 2 Signal Processing Laboratory (LTS4), Ecole Polytechnique Fédérale de Lausanne (EPFL), Lausanne, Switzerland; Universiti Tunku Abdul Rahman, MALAYSIA

## Abstract

Blood pressure (BP) is a crucial biomarker giving valuable information regarding cardiovascular diseases but requires accurate continuous monitoring to maximize its value. In the effort of developing non-invasive, non-occlusive and continuous BP monitoring devices, photoplethysmography (PPG) has recently gained interest. Researchers have attempted to estimate BP based on the analysis of PPG waveform morphology, with promising results, yet often validated on a small number of subjects with moderate BP variations. This work presents an accurate BP estimator based on PPG morphology features. The method first uses a clinically-validated algorithm (oBPM^®^) to perform signal preprocessing and extraction of physiological features. A subset of features that best reflects BP changes is automatically identified by Lasso regression, and a feature relevance analysis is conducted. Three machine learning (ML) methods are then investigated to translate this subset of features into systolic BP (SBP) and diastolic BP (DBP) estimates; namely Lasso regression, support vector regression and Gaussian process regression. The accuracy of absolute BP estimates and trending ability are evaluated. Such an approach considerably improves the performance for SBP estimation over previous oBPM^®^ technology, with a reduction in the standard deviation of the error of over 20%. Furthermore, rapid BP changes assessed by the PPG-based approach demonstrates concordance rate over 99% with the invasive reference. Altogether, the results confirm that PPG morphology features can be combined with ML methods to accurately track BP variations generated during anesthesia induction. They also reinforce the importance of adding a calibration measure to obtain an absolute BP estimate.

## Introduction

Cardiovascular diseases (CVDs) are the leading cause of death worldwide, accounting for 32% of lives taken. Persistently high blood pressure (BP), also known as hypertension, is the major risk factor for CVDs. In 2010, 31.1% of the adult population is estimated to be hypertensive, and this number will continue to increase due to population aging, lack of exercise and increased exposure to risk factors, such as overweight, unhealthy diet, tobacco and alcohol consumption [1]. The high prevalence of hypertension is partly explained by its asymptomatic aspect, as most individuals are not aware of the problem in early stages. Furthermore, BP can be rapidly altered by multiple external factors, including emotions, physical activity or medication. Consequently, the development of robust continuous BP monitoring is of utmost importance for the identification of atypical BP fluctuations, as well as early detection and management of hypertension and related CVDs. The gold standard method for BP monitoring is a pressure-sensing catheter inserted in an artery. Despite allowing precise and continuous monitoring, it is an invasive process with high risk of complications that cannot be deployed outside of the clinical environment. Accordingly, sphygmomanometry is considered as the conventional reference for non-invasive BP monitoring. Nevertheless, this approach measures BP with an inflatable cuff. It is therefore obstructive, causes discomfort and only allows intermittent measurements.

Motivated by these limitations, the focus has been shifted to the development of new non-invasive and cuffless BP monitoring technologies suitable for continuous measurement. One promising technique is based on photoplethysmography (PPG). PPG consists of a light-emitting diode (LED) and a photodetector. Such simple and cost-effective optical sensors can be easily integrated into wearable devices and are available in some smartphones. The PPG signal is sensitive to blood volume variations in the microvascular bed of tissues, and thus captures information related to cardiovascular parameters. Typically recorded with a pulse oximeter in clinical setting, it is commonly used in healthcare to monitor heart rate and blood oxygen saturation. Peripheral volumetric variations and BP are known to be correlated [2], which suggests that some characteristics of the PPG signal can be exploited to estimate BP. However, no clear mathematical formulation has been able to define the complex relationship between PPG signal and BP. Recent progresses in the study of PPG pulse morphology, also known as pulse wave analysis (PWA), have nevertheless highlighted a set of features playing a key role in the modeling of BP. The cardiac period, the systolic upstroke time and diastolic time, along with pulse widths at various amplitudes, have been first considered as informative features [3, 4]. Some frequency domain features have also been introduced, where amplitude and phase features are computed using the discrete Fourier transform method [5]. Moreover, characteristic points related to the second derivative of the PPG waveform add valuable information and have improved the BP estimation [6–8]. In this context, CSEM’s proprietary oBPM^®^ algorithm [9] has achieved good performance for systolic and mean BP estimation in patients undergoing general anesthesia [10], along with stability in its calibration [11]. This model is based on PWA and how arterial distensibility affects the pulse morphology. By tracking features related to arterial distensibility, it can indirectly track BP changes.

Despite these encouraging results, the main challenges in the application of PPG-based approaches for BP estimation consist in achieving precision and robustness that meet consumers’ and physicians’ expectations. This goal has not yet been fully achieved by previous works. Driven by the increased availability of larger labeled datasets, supervised machine learning (ML) seems to be a promising solution to learn a mapping between PWA-based features and BP values. The benefits of ML methods for this task have been investigated by multiple groups, with varying degrees of success. The choice of ML model typically depends on the amount of data available and the complexity of the task. The approaches commonly explored for BP estimation consist of linear and non-linear models. However, linear models might not be appropriate to represent the complex relationship between BP and PPG features. Their performances have been improved with other classical ML methods, such as support vector regression (SVR) [7, 12, 13], regression trees [14, 15], Gaussian process regression [8, 16], AdaBoost [17, 18] and feedforward neural networks (NN) [4–6]. Nevertheless, the reported outcomes should be interpreted with caution. First, some methods combine PPG and electrocardiography (ECG) as input. This involves simultaneous recordings of both signals, which can be inconvenient and might be less suitable for long-term wearable applications. The use of multiple sensors also increases the necessary preprocessing work. Second, the heterogeneity in the metrics and dataset used makes comparison and interpretation among different models difficult. The validation protocol is often based on datasets with low inter- and intrasubject BP variations. Consequently, the model generalization capability and ability to track significant BP changes are generally not verified. Another critical point is the calibration of the model, which helps to cope with the inter-subject variability of the PPG waveform. Although most published works did not mention a calibration procedure, they only divided samples between training and test sets but not subjects. Therefore, data from the same subject is used during both learning and evaluation of the model. Besides the increased risk of overfitting, this methodology has limited potential applications, as some subject’s data are required prior to the prediction. Finally, nearly all studies address mainly the estimation accuracy, without investigating the importance of the different features for the model performance, which could be highly insightful and contribute to a better understanding of the BP estimation problem and the generalization capabilities of the ML model.

The present work further develops the approach proposed in [16], which focused only on systolic BP (SBP) estimation and restricted the maximum time span since calibration to 2 minutes. We investigate the development of PPG-based ML models for BP monitoring with an in-depth physiological understanding. To this end, a model is built to extend the oBPM^®^ technology with effective ML methods. oBPM^®^ is used to preprocess the PPG signals and extract comprehensive physiological features. The first stage is the identification of important features from the full feature set. This feature selection step aims to define a subset of features that best reflects BP. In comparison with oBPM^®^ technology, the proposed approach comes from a data-driven perspective, especially due to its expanded initial feature set derived from PWA and automatic feature selection. The second stage defines a proper mapping of features into SBP and diastolic BP (DBP) values. Therein, the reduced feature sets are used for the investigation of higher-accuracy estimation models applying three different ML techniques; including Lasso regression, support vector regression and Gaussian process regression. Their performance is assessed based on the model ability to track acute BP changes as well as absolute BP estimates. All proposed models are compared to the clinically validated method of oBPM^®^ for benchmarking purposes and evaluated against the arterial line in the context of anesthesia induction. To investigate the importance of calibration information, which is one of the limitations of currently proposed PPG-based BP estimation methods, both calibration-dependent and calibration-free variants of the proposed algorithms are explored.

Beyond exploring different suitable ML methods to address the challenge of BP monitoring from PPG signal, the main contributions of this work are to investigate the features being essential in the modeling of BP and to provide an appropriate validation protocol, considering both the agreement with invasive reference as well as the ability to track large BP variations induced by anesthesia.

## Materials and methods

**Fig 1:**
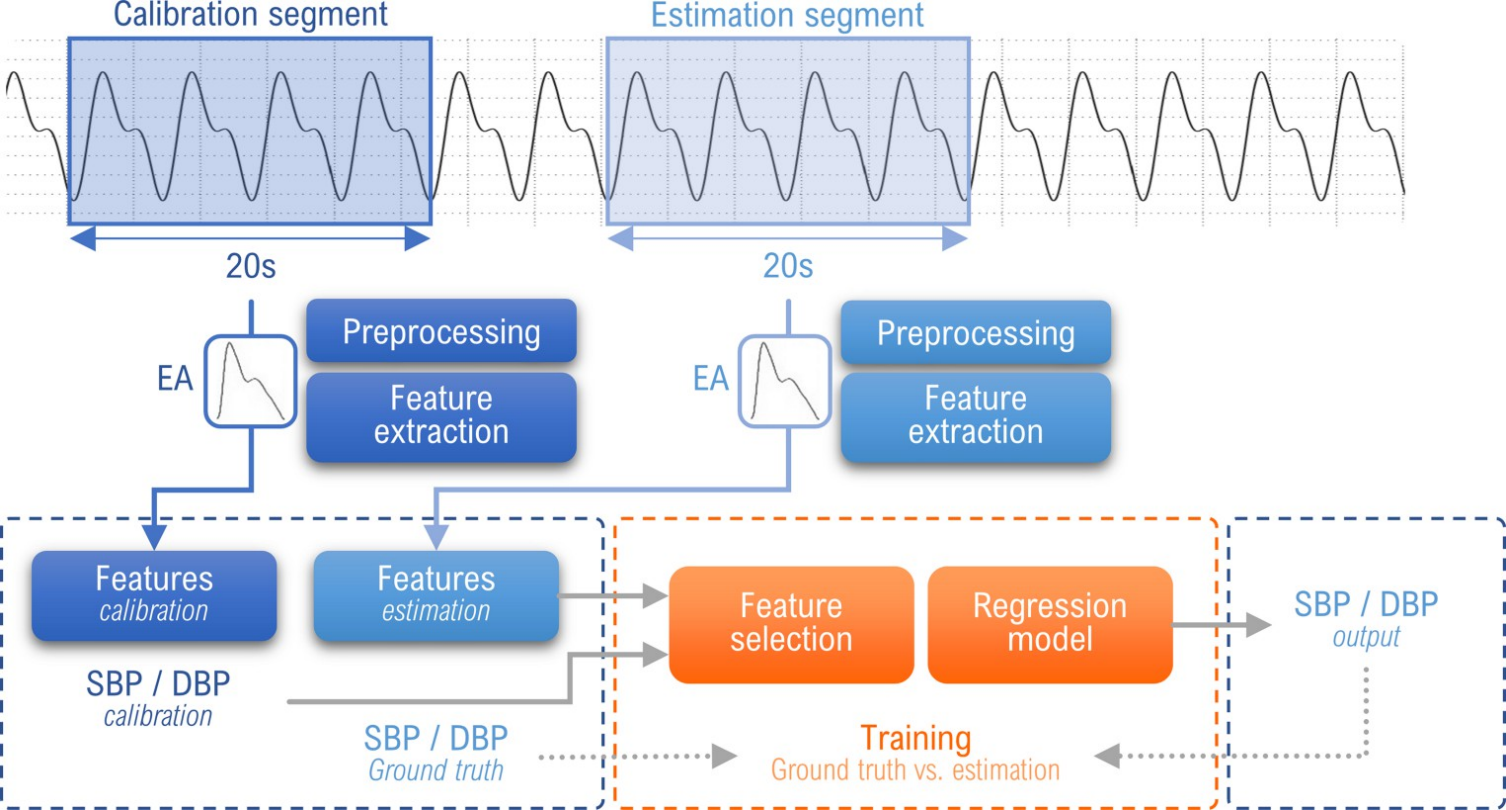
Schematic description of the approach with a calibration PPG-BP measure. The model combines oBPM^®^ pre-processing and feature extraction with ML methods for feature selection and BP estimation. After preprocessing and feature extraction, the model takes as inputs the features of the calibration and estimation segments with the references of the calibration. The references of the estimation segment are used as ground truth for the learning phase.

The proposed approach applies ML techniques to PWA-derived features computed by the oBPM^®^ technology. Some steps are particularly important in the development of a reliable data-driven model. This includes the extraction and selection of features related to BP, as well as the identification of a proper predictor to track BP changes with good generalization capability. The overall framework is illustrated in Fig 1 and the details of each step are explained in the following sub-sections. All analyses were performed using MATLAB R2020a (MathWorks, Natick, USA).

### Dataset

Training and testing data are from a clinical dataset collected in collaboration with the Lausanne University Hospital (CHUV) under approval of the local ethics committee (CER-VD, no. 327/15; NCT02651558 at ClinicalTrials.gov). It includes recordings of 40 adult patients undergoing general anesthesia for various surgical reasons (ear-, nose-, throat- or neurosurgery). Written informed consent was obtained from all subjects and data was anonymized. The patient demographics are summarized in Table 1. The raw PPG signals were obtained from a finger-clip sensor (Nellcor^TM^, Medtronic) via a custom datalogger. BP was synchronously monitored with an invasive catheter inserted in the radial artery.

**Table 1:** Demographic and BP characteristics.

Characteristics	Mean ± STD (Range) or count (%)
Age (y)	62.4 ± 12.7 (27–81)
Height (cm)	170.1 ± 9.89 (154–189)
Weight (kg)	72.6 ± 13.4 (46–102)
Gender, male	21 (52.5)
Hypertension	14 (35)
Per-subject SBP average (mmHg)	124.3 ± 24.8 (83.1–199.5)
Per-subject DBP average (mmHg)	63.0 ± 10.4 (42.1–86.4)
Per-subject MBP average (mmHg)	85.5 ± 15.5 (57.8–120.3)
Per-subject SBP std (mmHg)	18.7 ± 7.6 (4.8–33.4)
Per-subject DBP std (mmHg)	8.2 ± 3.0 (2.6–14.1)
Per-subject MBP std (mmHg)	12.3 ± 4.8 (3.2–22.1)

The recording sessions took place at induction of general anesthesia and lasted between 9 and 19 minutes per patient. Due to varying doses of anesthesia, strong BP variations over time were induced in this dataset. Within the study group, 14 patients had previously been diagnosed with hypertension. Overall, the per-patient average SBP varies between 83 and 200 mmHg, with a mean across patients of 124 mmHg. The per-patient average DBP is between 42 and 86 mmHg, with a mean of 63 mmHg.

To guarantee the reliability and robustness of the results, 80% of the dataset is used as learning data, while the remaining 20% is used for testing. Each subject is only assigned either to the learning set or the test set. To obtain sets with comparably balanced BP profiles, the separation is stratified according to the mean and standard deviation SBP and DBP values of the subjects.

### Pre-processing

After alignment of the PPG signal and arterial line signal based on cross-correlation, the pre-processing is done using oBPM^®^ technology. The raw PPG are filtered with two digital filters: a low-pass 3^rd^-order Butterworth filter with cut-off frequency of 15 Hz to remove high frequency noise, and a high-pass 3^rd^-order Butterworth filter with cut-off frequency of 0.5 Hz to remove the baseline. The PPG signals are then divided into 20 seconds segments with 50% overlap. After segmentation of cardiac cycles, PPG pulses are aggregated into an ensemble average (EA) pulse computed over each 20-second window. This process results in pulses with representative waveforms. A signal quality index of each individual pulse weights the averaging process. This value is calculated based on similarities with typical PPG waveforms and the neighbouring pulses [10]. The quality index (≥ 75%) as well as the reference variability (standard deviation/average ≤ 10%) over the 20-second window serve as exclusion criteria to remove unreliable pulses for further analysis. This results in a total of 2’741 EA pulses.

The reference SBP and DBP values are calculated from the invasive catheter signals as the median over each 20-second window and are used as ground truth values.

### Feature extraction

**Fig 2:**
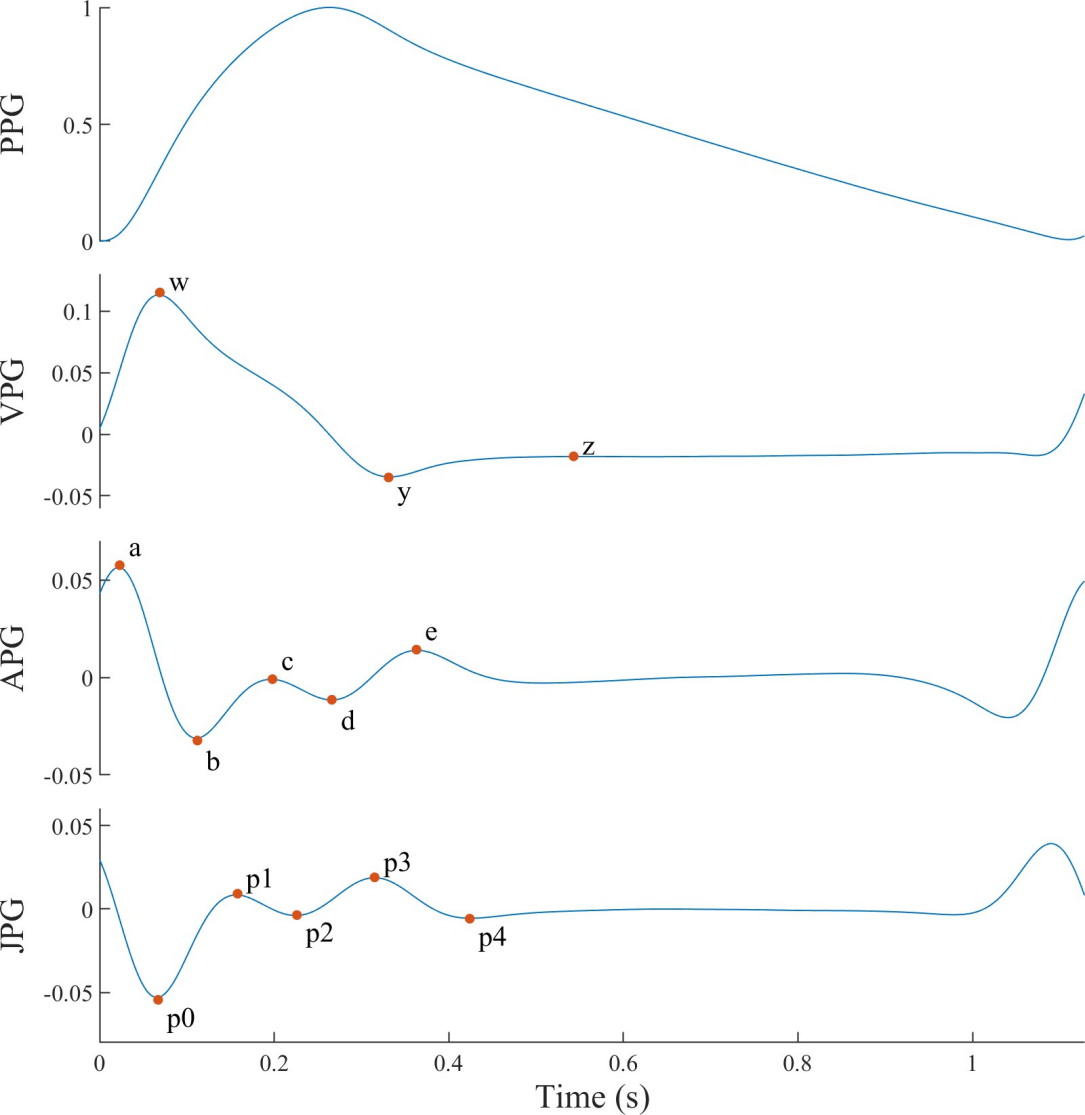
PPG derivatives with characteristic points. Illustration of a typical PPG waveform with its 1^st^, 2^nd^ and 3^rd^ derivatives, which are respectively known as velocity plethysmogram (VPG), acceleration plethysmogram (APG) and Jerk plethysmogram (JPG). The fiducial points of each derivative are also shown.

From the average pulses, domain knowledge can be used to create features that better represent the underlying problem. The PWA performed by the oBPM^®^ algorithm allows the extraction of time-related and amplitude-related features. The 1^st^, 2^nd^ and 3^rd^ PPG derivatives are computed to help the interpretation and understanding of the PPG waveform [19]. The 1^st^ derivative is also known as velocity plethysmogram (VPG), the 2^nd^ as acceleration plethysmogram (APG) and the 3^rd^ as Jerk plethysmogram (JPG) [20, 21]. This analysis follows from the fact that PPG waveforms share physiological similarities with the radial pressure pulse [22]. Morphological features are extracted based on characteristic points present on the average pulse and its derivatives. For the average pulse, it consists of mean arterial pressure (MAP), mean systolic blood pressure (MSBP), mean diastolic blood pressure (MDBP), end-systolic pressure (ESP), systolic pressure-time index (SPTI), diastolic pressure-time index (DPTI), sub-endocardial variability ratio (SEVR), perfusion index (PI) and augmentation index corrected for a heart rate of 75 bpm (AIx75). For the PPG derivatives, the time and the amplitude of the characteristic points illustrated in Fig 2 are also considered. Further details on the PWA and the extracted features are described in [10, 23].

PWA-derived features are combined with some personal information of the patients, including age, weight, height and gender (Table 1). These are important confounding factors, known to affect BP, as well as the PPG waveform. Due to inter-subject variability of the pulse morphology, adding prior information regarding personal details might improve the model performance and help to adjust to different subjects.

### Calibration approach

The BP estimation model usually depends on some initial calibration process, either between individuals or over time. This can correct possible baseline drifts, and significantly reduce the bias of the estimation. Therefore, the first learning strategy includes an initial calibration PPG-BP measure to correct this offset. For ambulatory BP monitoring, a cuff measurement at the doctor’s office can be used to perform the calibration. As the present study took place in the operating room, an invasive reference measure is used instead. The flow chart of the approach is illustrated in Fig 1. For each sample, the calibration PPG segment is always taken along the same recording as, and preceding, the estimation segment. For each recording, we start by taking the first PPG segment as calibration segment and consider all the subsequent segments as estimation segment. We iteratively move the calibration segment along the recording and repeat the pairing process. By considering all combinations, we increase the number of samples to a total of 95’833. After preprocessing the PPG signal, features are extracted from each segment, as further described in previous section. Each datapoint combines the PWA-derived features extracted from the calibration segment with the corresponding reference BP values (SBP, DBP and mean BP), the PWA-derived features of the estimation segment and the demographics. The reference BP values of the estimation segment serves as ground truth during the model training. The time span between the calibration and estimation segments is between 10s and 1119s, with an average of 255s (Table 2).

**Table 2:** Characteristics of calibration.

Characteristics	Mean ± STD	Range
ΔT (s)	254.8 ± 187.2	(10.0–1119.0)
ΔSBP (mmHg)	-9.7 ± 26.4	(-116.3–110.8)
ΔDBP (mmHg)	-3.1 ± 11.7	(-50.4–53.6)
|ΔSBP| (mmHg)	20.5 ± 19.2	(0–116.3)
|ΔDBP| (mmHg)	9.0 ± 8.1	(0–53.6)

Calibration is one of the main limitations of most PPG-based BP estimation methods, as requiring a measure with an approved standard complicates real-world applications. Therefore, we also carry out experiments without calibration procedure, with a model taking the PWA-derived features from the estimation segment together with patient demographics as input. This results in 2’741 samples available. This step assesses whether a calibration-free model allows prediction of relatively accurate BP and evaluates to which extent calibration is necessary.

### Feature selection and feature relevance analysis

No explicit set of PPG features has been defined to be directly related to BP, and research is still ongoing. As described in the feature extraction subsection, numerous features characterizing the morphology of a PPG pulse can be extracted. A large feature set increases the model complexity and therefore makes it less interpretable. Understanding the decision process could help to gain new insights on the relationship between PPG and BP signals. Therefore, it is necessary to remove irrelevant and redundant features to obtain an optimal feature subset that best describes the output variable. In addition to reducing the model complexity, this process also decreases the training time and the risk of overfitting.

In this study, Lasso (least absolute shrinkage and selection operator) regression [24] is applied to define a subset of relevant features. This embedded method has a feature selection integrated as part of the model learning process. This linear model simultaneously achieves feature selection and regression to improve the prediction accuracy and model interpretability. By regularization with an L1 norm, it imposes sparsity constraints on the regression coefficients. In more details, we start by training Lasso on the full feature set, including PWA-derived features and personal information. A relevance score of the selected features is computed based on the permutation feature importance method [25]. This metric shows how much the estimation error is increased when a given feature is randomized. It ranks features according to their effect on the model prediction. In addition to being model agnostic, this method does not require complex mathematical computation or model retraining. Furthermore, it considers both individual feature effect as well as their interaction on the model performance. The relevance score is obtained with the procedure described below.

1. The values of a given feature across all samples in the test set are randomly permuted, resulting in a modified version of the test set.
2. The model estimates the SBP and DBP taking as inputs the modified test set.
3. The relevance of the disrupted feature is computed as the relative increase in the standard deviation of the error (STDE) between the permuted and the original test set.
4. The process is repeated 100 times per feature to compute an average relevance over random permutations.

This analysis helps to understand the importance of the calibration measure. Different aspects are investigated. The calibration and estimation segments might not have the same impact on the estimation. As their features are concatenated and not subtracted before the selection process, the most relevant features of the estimation segment might not be similar to those of the calibration segment. Furthermore, the feature selection procedure is repeated for the calibration-free variant. Therefore, the calibration-dependent and calibration-free variants might have a different selection of features.

### Regression models

The second objective of this work focuses on the ability of various ML methods to provide absolute *BP* estimation. Due to the continuous nature of the data, we use regression-based ML approaches to translate the selected features into the corresponding SBP and DBP values. The different regression models are then trained and optimized taking as input those selected features. Choosing a suitable ML model is always a trade-off between multiple characteristics, including memory usage, speed, prediction accuracy, model complexity and interpretability. This last point is particularly important in healthcare applications and has led to the following choice of ML models:

1. Lasso regression (Lasso) [24]. Linear models are generally a good first approach as being easily interpretable and well-understood. Lasso is of particular interest as its regularization term helps to prevent overfitting. Hyperparameter: weight of sparsity-imposing term *λ*. Optimized using a 10-fold cross-validation. Folds are stratified by subjects.
2. Support vector regression (SVR) [26]. This method is worth considering as it gives the flexibility to define the model error tolerance. Hyperparameters: kernel scale, box constraint of *α* coefficients, width of *ε-*insensitive band. Optimized using 10-fold cross-validation and Bayesian methods [27].
3. Gaussian process regression (GPR) [28]. This method adds a probabilistic perspective and can capture the model uncertainty. Hyperparameter: noise standard deviation of the Gaussian process model *σ*. Optimized using 10-fold cross-validation and LBFGS algorithm (Limited-memory Broyden-Fletcher-Goldfarb-Shanno) [29].

### Evaluation method

The accuracy and precision of the proposed algorithms in providing absolute BP are evaluated by comparing the estimates from the non-invasive method to the invasive reference. Many studies follow the guidelines of ISO 81060–2:2018 norm [30]. This criterion requires a mean error between estimated and reference BP values (ME) not greater than ±5 mmHg, as well as a STDE not greater than 8 mmHg. Although developed as a universal standard for sphygmomanometers with strict recording protocols, in absence of applicable norms, it is commonly taken as point of comparison for non-invasive cuffless BP estimators as well, but should not be considered to assess model accuracy. The proposed models are evaluated against the invasive reference in terms of ME and STDE in mmHg. Comparing such metrics between train and test samples provides insight regarding the generalization capability. The model should not overfit the train data and generalize well to unseen data, meaning achieving a comparable STDE in the test set. This allows the model to adapt to different subjects and slightly different recording protocols.

For benchmarking purposes, we also optimize the model parameters of the clinically-validated oBPM^®^ algorithm [9] on the training set and test it alongside all proposed models. As control case, a “flat model” is also included in this study [31]. By assuming no BP variation over time, this simple model estimates the BP to be equal to the calibration reference. In the experiments without calibration, this model outputs the average BP values over the training set. In addition to a baseline performance, its STDE provides helpful information regarding the BP variations in the dataset.

However, the standard norm does not allow a proper evaluation of the accuracy in tracking short or long-term BP variations within an individual [31]. Therefore, another objective of the present study is to evaluate the trending ability of the best proposed PPG-based algorithm against the invasive reference. The dataset used includes rapid and large BP variations over time due to administration of anesthetic and vasoactive drugs. It is therefore of particular interest to study this performance aspect. The trending ability of the selected ML model in providing absolute BP changes is evaluated using four-quadrant plots [32] and polar plots [33]. All possible BP changes of at least 20% [34] are identified from the reference (ΔSBP_inv_ and ΔDBP_inv_), along with the corresponding BP changes assessed by the PPG-based method (ΔSBP_PPG_ and ΔDBP_PPG_). Such changes are considered acute by clinicians and require an action on their part. Based on the four-quadrant plots, the concordance rate and the Pearson correlation coefficient between invasive and non-invasive approaches are computed. The concordance rate (CR) represents the proportion of (ΔBP_inv_, ΔBP_PPG_) paired values with similar direction of change. Whereas the four-quadrant plots focus on the direction of BP change, the polar plots add information relative to the amplitude of BP change. In such plots, the polar radius reflects the average amplitude of change of invasive and PPG-based methods ((Δ*BP_inv_*+Δ*BP_PPG_*)/2) and the polar angle represents the drift from the identity line. The angular concordance rate at ±30° is then calculated. It reflects the amount of data lying within the upper radial limits of ±30°, as defined in [33]. The angular bias and standard deviation of the polar angle are also assessed.

## Results

### Feature relevance analysis

**Fig 3:**
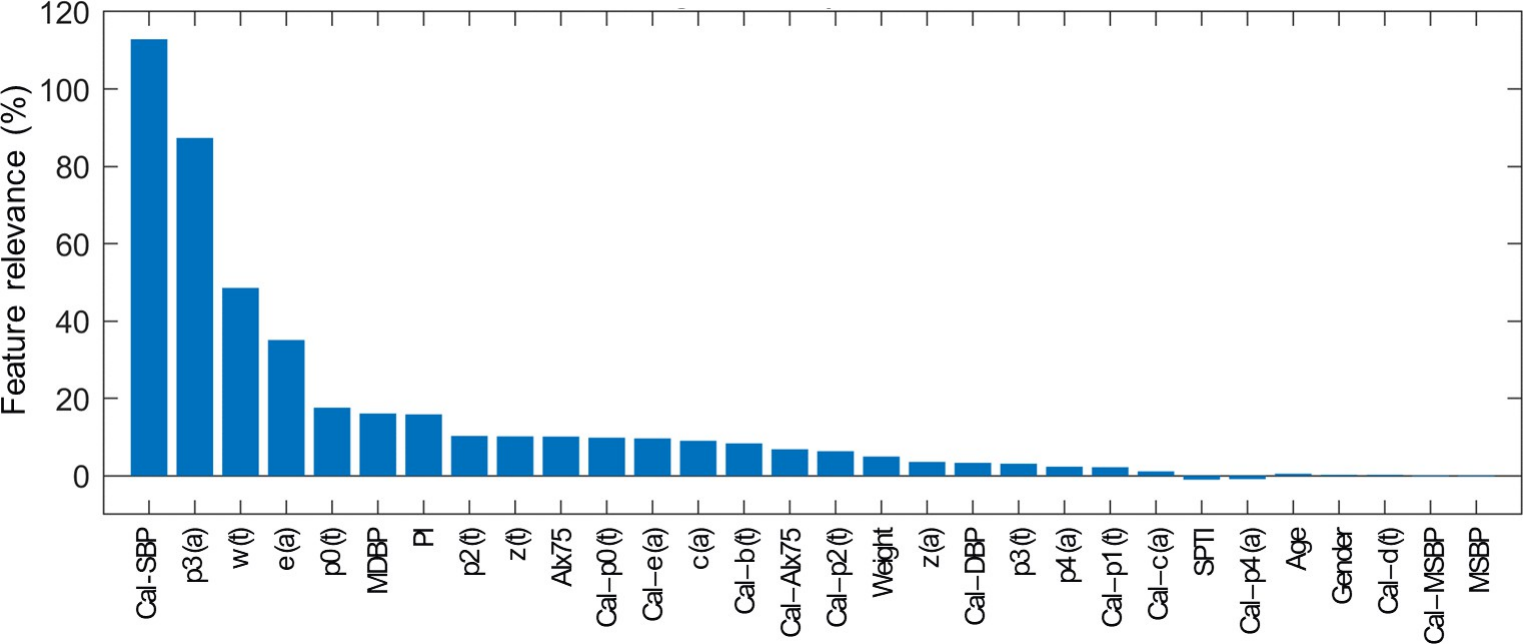
Feature relevance scores for SBP estimation model with a calibration PPG-BP measure. Features from the calibration segment are indicated by the prefix cal. The reference value obtained the highest relevance. The following were PWA-derived features from the PPG waveform, as well as time (t) and amplitude (a) of characteristic points from its derivatives (VPG, APG and JPG). Personal information, such as weight, obtained a low relevance score. The results were alike for DBP.

**Fig 4:**
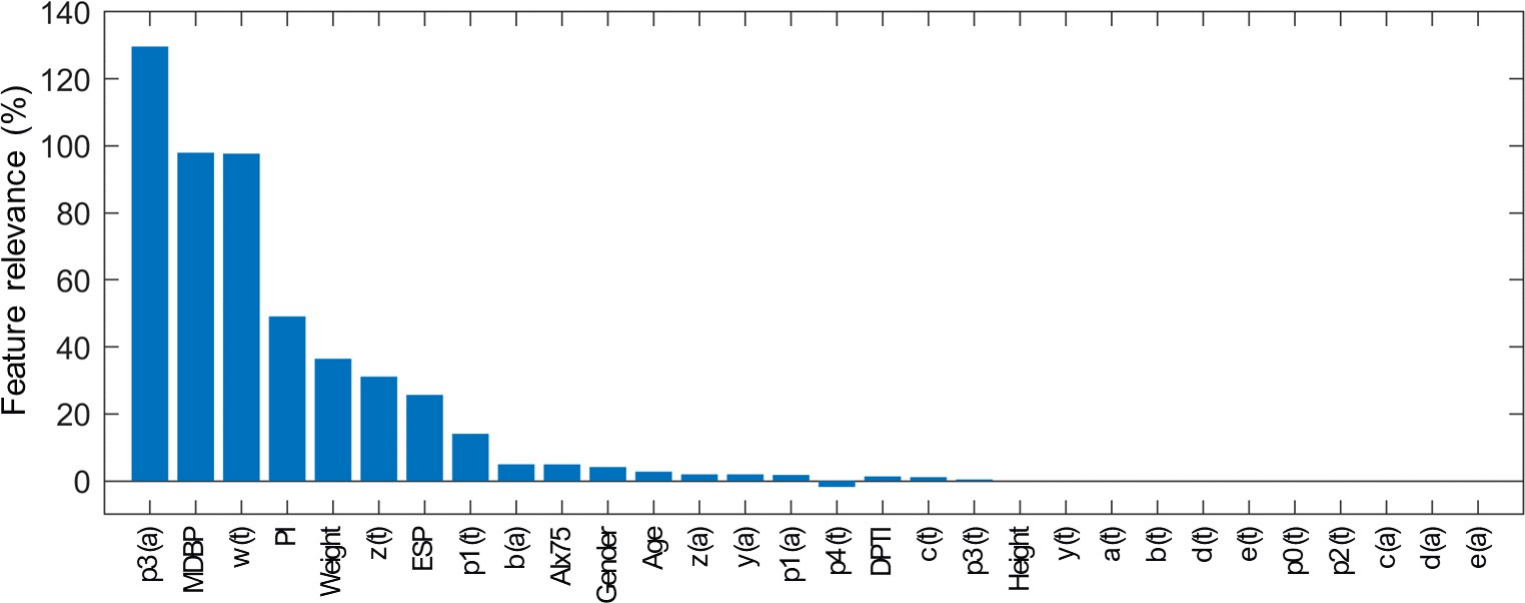
Feature relevance scores for SBP estimation model without a calibration measure. PWA-derived feature from the PPG waveform, as well as time (t) and amplitude (a) of characteristic points of its derivatives (VPG, APG and JPG) were of high relevance. Personal information had more weight than for the model with a calibration measure.

During the feature selection process and when learning requires a calibration measure, Lasso picked 35 and 24 features for SBP and DBP, respectively, while only 19 features are selected for the cases without calibration for both SBP and DBP. There are some similarities in the feature sets of the different BP estimators, with slight variations in their order of importance. Therefore, the results of the feature relevance analysis are only displayed for SBP in Figs 3 and 4. For BP estimation with a calibration measure, the reference value of the calibration segment is detected as the most important feature. The following features with high relevance are mainly PWA-derived features, including: features from the PPG, such as perfusion index (PI) and heart rate-corrected augmentation index (AIx75), or characteristic points of its first (VPG), second (APG) and third (JPG) derivatives. As predicted, the features selected from the calibration and estimation segments are related, but not identical. Furthermore, features from the estimation segment generally come first. Highly important features from the estimation segment are also present with a high relevance score in the calibration-free variant. Personal information obtains a low feature relevance score in calibration mode. The first one is the subject’s weight appearing in 17^th^ rank (Fig 3). However, when no calibration PPG-BP measure is given, personal information gets a higher relevance score, with the subject’s weight in 5^th^ rank (Fig 4).

### BP estimation

Tables 3 and 4 summarize the performance of the different ML models for SBP and DBP estimation with a calibration measure in terms of ME and STDE in mmHg. The first observations are drawn for SBP estimation in calibration mode, with a particular focus on the STDE in the test set. All the regression models implemented outperform the control case (flat model). The reduction in STDE is approximately 60%. Furthermore, the three proposed ML models are superior to the oBPM^®^ algorithm, with a reduction in STDE of 24–29%. The STDE of Lasso and SVR models on the train set is slightly smaller than on the test set, suggesting a low overfitting. On the contrary, even with hyperparameter optimization and a restricted feature set, GPR demonstrates a stronger overfitting effect. The observations are quite similar for DBP estimation with calibration. All proposed models are about 40% better than the flat model according to the STDE. However, they perform similarly to the oBPM^®^ algorithm, with a reduction in STDE of maximum 5%. By comparing the STDE of the train and test sets, we observe a larger overfitting effect with the three proposed ML models and oBPM^®^ for DBP than achieved for SBP.

**Table 3:** SBP estimation performance in mmHg.

	Train		Test	
Model	ME	STDE	ME	STDE
Lasso	-0.03	9.01	-0.87	10.77
SVR	-0.37	9.11	-2.27	9.99
GPR	-0.18	7.95	-2.46	10.28
oBPM ^(a)^	0.05	14.59	-2.16	14.24
Flat model ^(b)^	9.33	26.30	10.60	26.46

(a) Conventional oBPM^®^ model, with parameters optimized on training set.

(b) Naïve model, with calibration value as BP estimate.

**Table 4:** DBP estimation performance in mmHg.

	Train		Test	
Model	ME	STDE	ME	STDE
Lasso	0.00	4.41	-1.31	7.62
SVR	-0.22	4.49	-1.68	7.24
GPR	-0.03	3.73	-0.84	6.88
oBPM^a^	0.02	5.42	-0.60	7.21
Flat model^b^	2.78	11.34	3.79	12.58

^a^Conventional oBPM^®^ model, with parameters optimized on training set.

^b^Naïve model, with calibration value as BP estimate.

**Fig 5:**
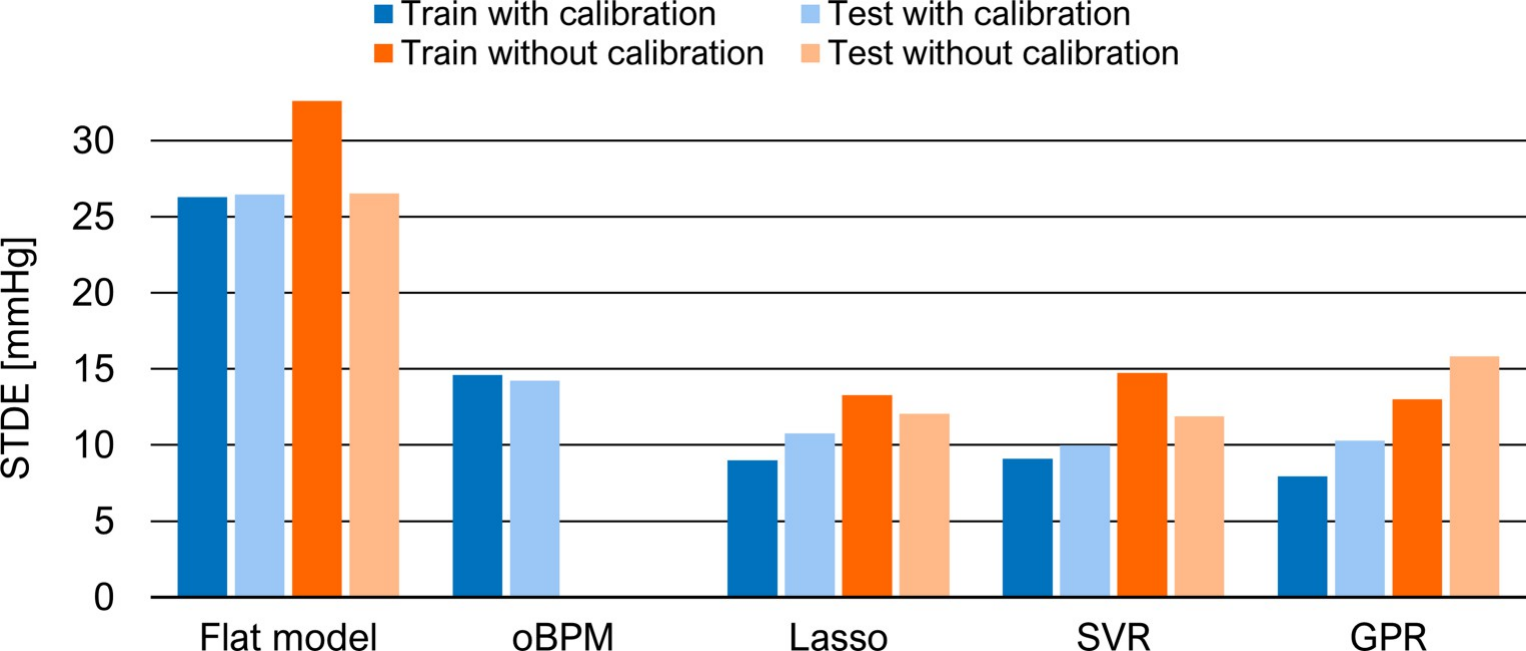
Performance of different ML models in term of STDE in mmHg for SBP estimation with and without calibration.

**Fig 6:**
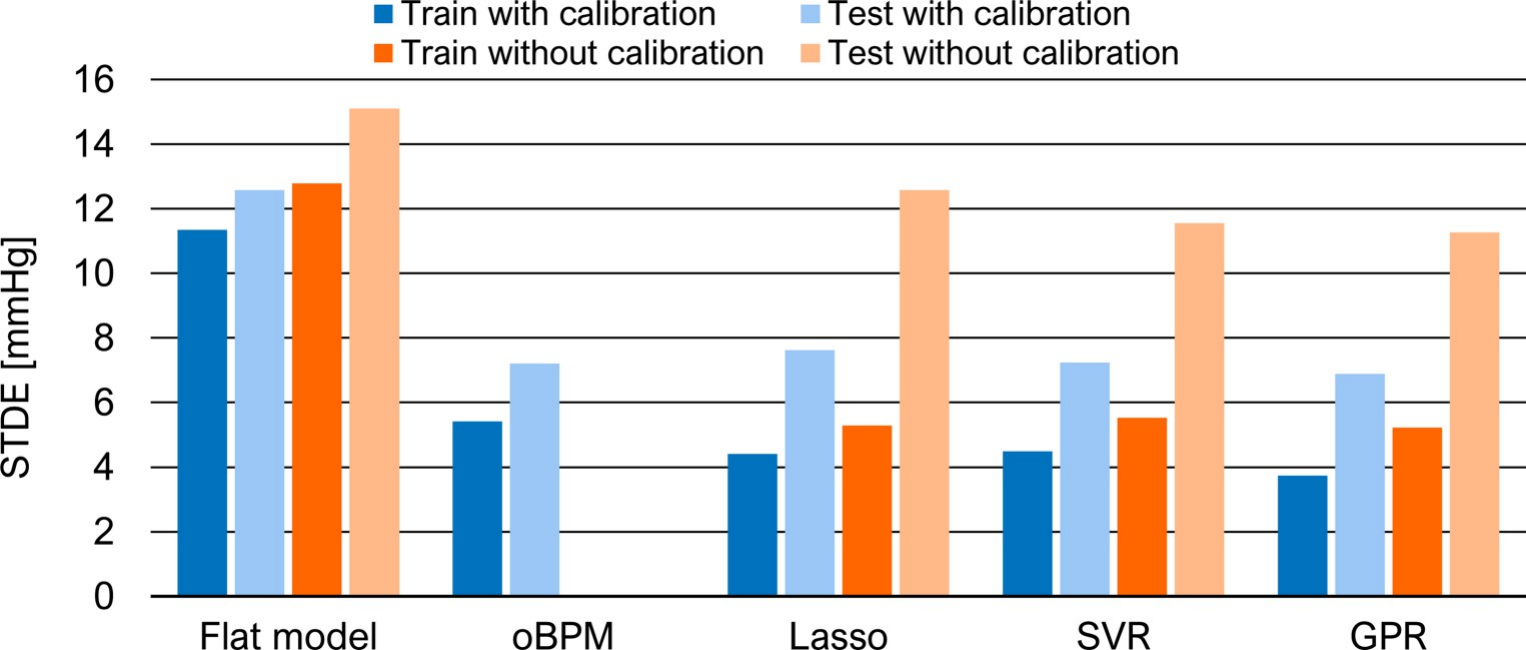
Performance of different ML models in term of STDE in mmHg for DBP estimation with and without calibration.

**Fig 7:**
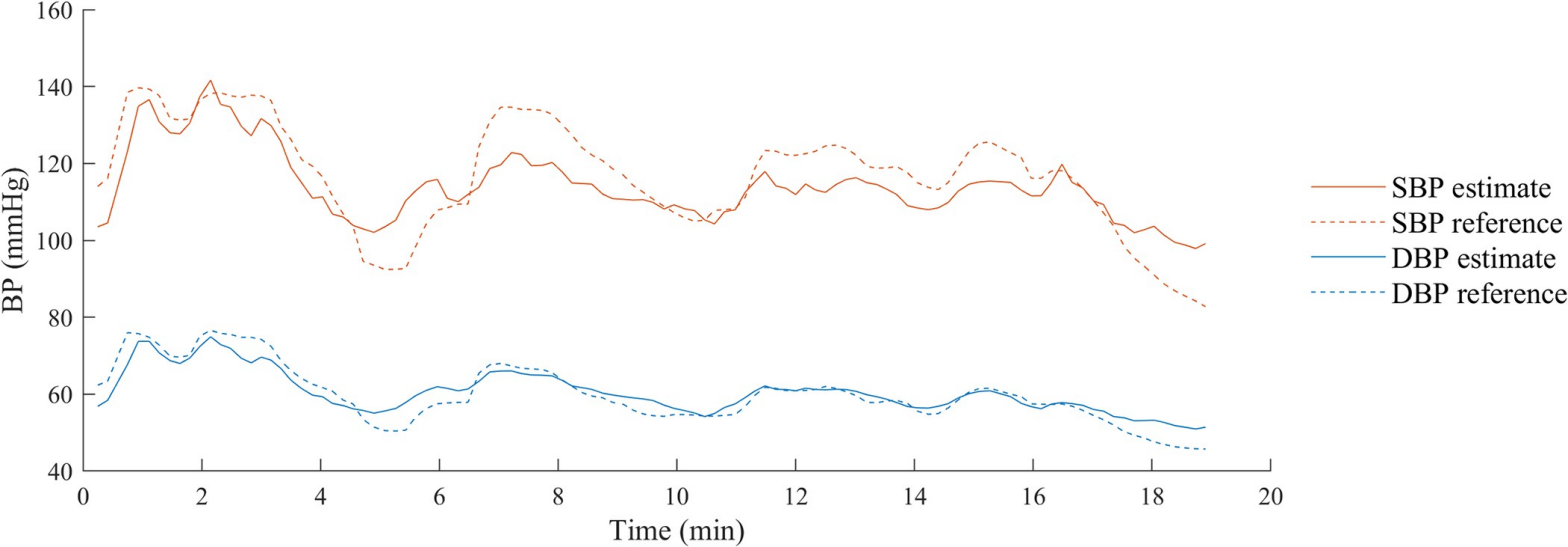
Temporal evolution of invasive reference and PPG-based BP estimation in a patient. Orange corresponds to SBP, while blue to DBP. The PPG-based BP estimations are shown by the solid lines. The invasive reference measurements are represented by the dashed lines.

Additionally, Figs 5 and 6 allow a better visualization of the STDEs and a comparison between calibration and calibration-free models for SBP and DBP, respectively. Once more, the performance is comparable between the different ML methods in calibration-free mode. For SBP estimation, the proposed ML models are superior to the control case, with a reduction in STDE between 40% and 55%. The addition of a calibration PPG-BP measure to the model reduces the STDE on the test set by more than 10%. For its simplicity, reduced overfitting effect and comparable performance, Lasso regression model with a calibration PPG-BP measure is chosen for the remaining part of the study. To better understand the BP dynamic during anesthesia induction, Fig 7 shows an example of the temporal evolution of the invasive reference and BP estimation with this model in a patient. In addition to the cohort-wise performance given in Tables 3 and 4, we also provide subject-wise results of this model on the test set in Table 5. Furthermore, we compare the performance of this model to related works in Table 6. Since it is a challenging task due to the heterogeneity in validation protocol, BP variability and calibration procedure, we only select works using the same evaluation metrics, i.e. ME and STDE. In particular, this comparison shows that the proposed work is based on a dataset including a larger BP variability. Our approach also has the most suitable calibration procedure for potential applications. Among the works mentioned, it is the only model which does not require to be retrained with new subject data and only depends upon a single spot PPG-BP measurement.

**Table 5:** Subject-wise BP estimation performance on the test set in mmHg.

	Mean ± STD	
	SBP	DBP
Subject ME	-0.67 ± 7.21	-0.82 ± 7.32
Subject STDE	9.06 ± 3.41	4.23 ± 1.33

**Table 6:** Table of comparison with related works in mmHg.

		BP	SBP		DBP	
	Model	Mean ± STD or (Range)	ME	STDE	ME	STDE
Xing and Sun [5] (2016)	NN	80 ≤ SBP ≤ 180	0.06	7.08	0.01	4.66
		DBP ≥ 20				
Gaurav et al. [6] (2016)	NN	-	0.16	6.85	0.03	4.72
Khalid et al. [14] (2018)	Regression tree	SBP: 109 ± 17	-0.1	6.5	-0.6	5.2
		DBP: 59 ± 9				
Hasanzadeh et al. [18] (2020)	AdaBoost	SBP (90–180)	0.09	10.38	0.23	4.22
		DBP (50–90)				
This work	Lasso	SBP (63–228)	-0.87	10.77	-1.31	7.62
		DBP (35–121)				

**Fig 8:**
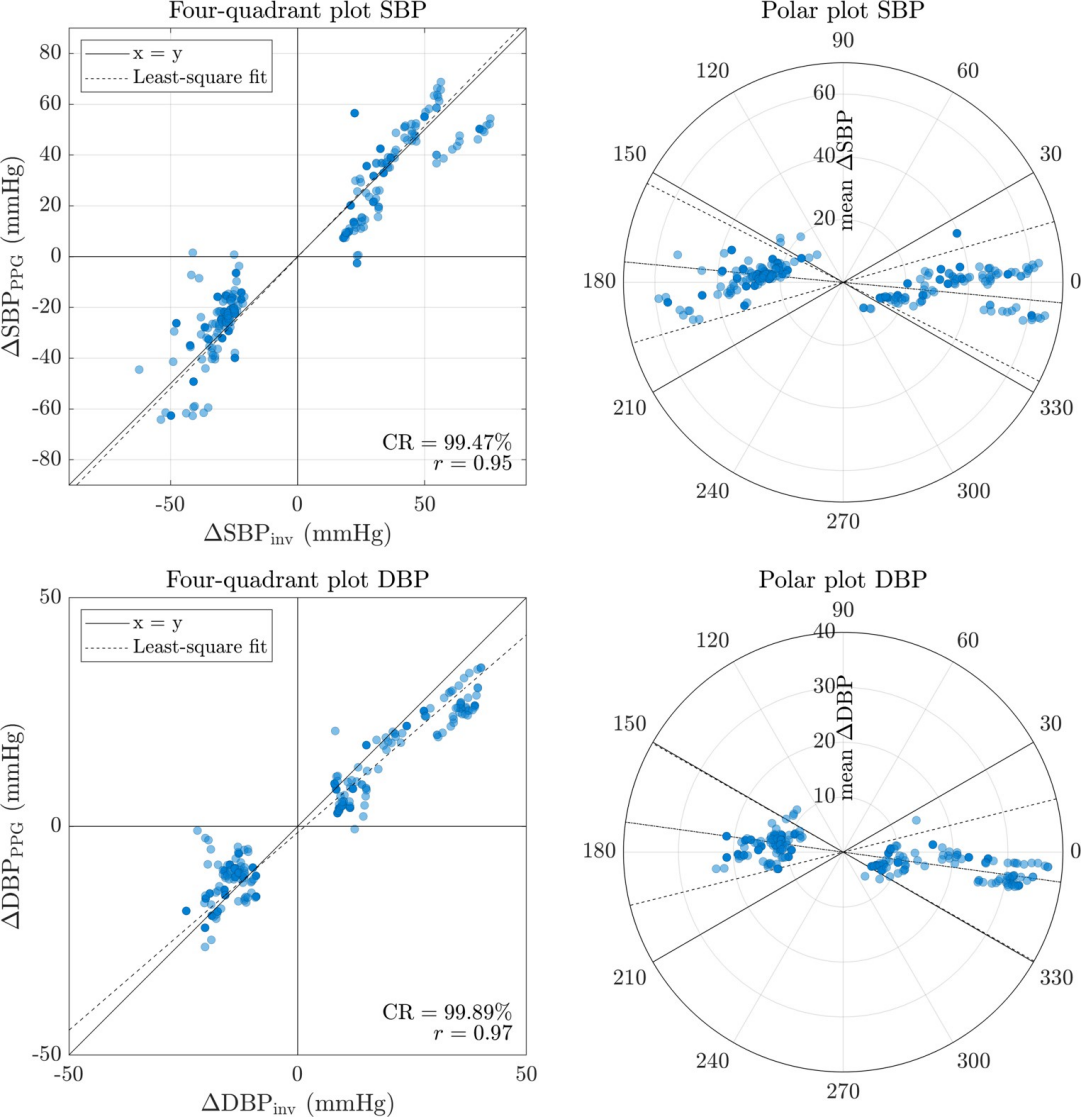
Trending ability. Four-quadrant plots (on the left) and polar plots (on the right) demonstrating the trending ability of PPG-based approach with Lasso regression against the invasive reference for rapid changes in SBP (top) and DBP (bottom) occurring over a time span of 3 min. The solid lines correspond to the ±30° upper radial limits and define the area in which more the 95% of the data points should lie. The angular bias is represented by the dashed-dotted line. And the 95% confidence interval by the dashed lines.

The trending ability of the PPG-based method against the invasive reference is assessed from the four-quadrant plots and polar plots. The results illustrated in Fig 8 are for rapid SBP and DBP changes occurring over a time span of 3 minutes with Lasso regression model. The greatest number of the (ΔBP_inv_, ΔBP_PPG_) paired values lies within the two concordant quadrants of the four-quadrant plots, located in the lower left and upper right positions. This results in a concordance rate of 99.47% for SBP and 99.89% for DBP. The Pearson correlation coefficient indicates a strong correlation between the invasive and non-invasive changes (0.95 for SBP and 0.97 for DBP). Similar observations are made for slower changes occurring over a time span greater than 3 minutes. In such conditions, the concordance rate is of 94.68% for SBP and 97.63% for DBP, and the Pearson correlation coefficient was 0.93 for SBP and 0.95 for DBP. The polar plot adds information regarding the amplitude of the BP changes. The angular concordance rate at ±30° computed for rapid changes is 97.97% for SBP and 99.35% for DBP. 95% radial limits of agreements of [-26.77°, 16.07°] for SBP and [-29.73°, 14.02°] for DBP are obtained. And the angular error (mean ± std) is -5.35±10.91° for SBP and -7.85±11.14° for DBP.

## Discussion

In this study, we have investigated the potential of data-driven models to address the challenge of BP estimation from PPG signal in the context of general anesthesia induction. Beyond exploring different suitable ML methods, we have examined the features being essential in the modeling of BP and evaluated the model ability to track large BP changes.

### Feature relevance analysis

In the literature, PPG-PWA has mainly been used to assess cardiovascular parameters, such as arterial stiffness or vascular tone. Both are relevant predictor of cardiovascular events and related to BP [23, 35]. Part of this work investigates which features derived from such PPG-PWA strongly contribute to the task of BP estimation. The results show the complexity of this task, with the selected features being related to phenomena indirectly related to BP. Whether in calibration or calibration-free mode (Figs 3 and 4), the main PWA-derived features are similar. The feature relevance analysis reveals the importance of characteristic points from the original PPG, as well as from the first (VPG), second (AGP) and third (JPG) derivatives. The features based on the original PPG waveform that particularly stand out from this analysis are the augmentation index and the perfusion index. The augmentation index and its value corrected for a heart rate of 75 bpm (AIx75) are based on the onset of the backward reflected wave. This index is related to pulse wave velocity and arterial stiffness [23, 36]. Older patients have stiffened arteries and therefore a faster pulse velocity. This results in an early return of the reflected wave and raised SBP. The perfusion index (PI) represents the ratio of pulsatile to non-pulsatile blood flow in peripheral tissue and is known to correlate relatively well with the vascular tone. Hypertension is associated with an impaired endothelial function and an increased vascular tone [37]. The derivatives of the PPG waveform are computed to facilitate the detection of inflection points and to help the interpretation of the PPG waveform [19]. The first derivative of the PPG is rarely used in literature. However, features such as the maximum slope of upstroke can be derived from it and appear in the relevance analysis. In contrast, the second derivative has been used more broadly. The different waves of the second derivative pulse were defined in [19, 38], and computing the ratios of each wave to the first one results in indicators of arterial stiffness. Therefore, the presence of features derived from the second derivative in the relevance analysis was expected and is consistent with other studies [7]. To the best of our knowledge, only the work in [39] mentioned the use of features from the third derivative of the PPG waveform. Nevertheless, its approach combined ECG and PPG signals to achieve BP estimation. A novelty of the proposed approach is to incorporate the third derivative of the PPG and such features appear to obtain a good relevance score.

Overall, the relatively large number of selected features further demonstrates the complexity of the biophysical mechanisms underlying the measure of BP. It might also reflect the limited quantity of data available. Lasso regression is a linear approach and thus might be insufficient to fit the optimal, potentially simplest relationship between the features and the reference BP values. However, few non-linear features selection methods such as GPR were previously considered and did not perform substantially better than Lasso [16].

### Calibration

The results of the feature relevance analysis also confirm the importance of the calibration to obtain an absolute BP estimate. Each individual physiology impacts the relation between PPG waveform and BP. In the calibration approach, this aspect is minimized through the initial PPG-BP measure. This is notably revealed with the reference BP value receiving the highest relevance score. Additionally, some PWA-based features from the calibration segment are selected, although typically getting a lower relevance score than those from the estimation segment. Nevertheless, different features are selected from the calibration and estimation segments. This indicates that simply subtracting their features before the feature selection process would not be sufficient for an accurate BP estimation and thus supports the choice of the proposed architecture. When comparing the calibration and calibration-free approaches in Figs 5 and 6, the results show that, as expected, the initial PPG-BP measure helps to improve the algorithm performance in providing absolute BP. For instance, it allows for a reduction in STDE in the test set of approximately 10% for SBP estimation with Lasso and SVR models. The calibration-free approach might be too limited to fully characterize the inter-subject variability. Personal information, such as weight or gender might not be sufficient to reduce the impact of individual physiology.

### Validation of selected features

Although the selected features are shown to have a physiological consistency with cardiovascular parameters related to BP changes, the chosen set needs to be further validated by examining the model accuracy in providing absolute BP. Whether with or without a calibration PPG-BP measure, the performance of the three ML methods is comparable and gets closer to the commonly used standard (5±8 mmHg), although not applicable to cuffless BP estimators. As previously mentioned, their STDE on the test set is consistently lower than for the flat model, and mostly below the oBPM^®^ model, which pointed out that the expanded feature set of oBPM^®^ technology could improve performance. Overall, the results are promising and validate that the chosen feature set plays a key role in the modeling of BP.

### Model selection

Choosing a ML method for a particular task is always a question of trade-off between multiple characteristics, including memory usage, speed, predictive accuracy and interpretability. The limited overfitting effects observed for Lasso and SVR suggests that these methods might have a better generalization capability on new data than GPR. However, due to its higher complexity, GPR might suffer from the limited amount of data available here. Furthermore, even though GPR adds a probabilistic perspective, it is the method of the list with the highest computational cost, which could cause trouble for embedding into wearables. The interpretability of a model is inversely proportional to its complexity. Being a linear regression model, Lasso is more interpretable than SVR and GPR. This particularity might help to understand the decision process and some aspects of the underlying relationship between PPG morphology and BP values. For its interpretability and limited overfitting effect, the calibration approach using Lasso regression is used for the rest of the analysis. Its performance on the test set is of -0.87±10.77 mmHg for SBP and -1.31±7.62 mmHg for DBP.

### Trending ability

The trending ability analysis conducted helps to assess the model ability to track acute BP changes. The four-quadrant plot and polar plot are used for this purpose. By analogy with the work on non-invasive cardiac output monitoring in [33], a good trending ability can be claimed when the concordance rate and the angular concordance rate at ±30° are greater than 90–95%. With a dataset recorded during surgical interventions with general anesthesia induction, this analysis is mainly focused on rapid (≤ 3 minutes) and acute (≥ 20%) BP changes that might be undetected by the cuff and lead to neglected hypo- or hypertension. In this study, more than 99% of the fast BP changes assessed with the PPG-based method have a direction of change consistent with the reference invasive method. The aforementioned criterion has also been fulfilled for slower BP fluctuations, occurring over a time span greater than 3 minutes (CR > 94%). Significant BP changes from the PPG-based method are also strongly correlated with those measured with the arterial line (Pearson correlation coefficient ≥ 0.95 for rapid changes and ≥ 0.93 otherwise). Whereas the four-quadrant plot mainly provides information on the agreement of invasive and PPG-based methods in terms of direction of change, the polar plot adds insights about the amplitude of the changes. The angular concordance rate at ±30° for rapid BP changes is above the criterion expected for good trending ability (> 97%). The 95% radial limits of agreement help to quantitatively assess the precision. For SBP and DBP rapid changes, the results end up within the suggested ±30° upper radial limits [33]. Altogether, the previous observations confirm the good trending ability of the proposed PPG-based method and suggest that it is a particularly promising tool for tracking significant BP changes, which are known to be problematic in the clinical environment.

### Potential applications and limitations

Although the present study was conducted in a well-defined clinical setting, the low requirements for PPG signal acquisition broaden the potential application of the proposed PPG-based BP estimation method to diverse contexts, both inside and outside of operating room. The finger-clip sensor used for the recording of PPG signal is already part of the equipment in the operating room for monitoring vital parameters, such as oxygen saturation and heart rate. For patients without an arterial line, the proposed model might help the anesthesiologists to quickly detect unexpected BP changes between intermittent cuff inflations and to stabilize the patient. This approach could also be used as a solution trigger oscillometric cuff measurements automatically when significant BP fluctuations occur (≥ 20%). However, PPG sensors are increasingly integrated into wearable devices, such as smartwatches. This opens up the possibilities of ambulatory BP monitoring using a PPG-based method, an application that should be further investigated.

Furthermore, this study confirms that the dependence on calibration remains a key point in the development of non-invasive, cuffless and continuous BP monitoring solutions. The choice of calibration process will also have an impact on the performance. Our model is designed to correct the offset with an initial PPG-BP measure. This process is well representative of a possible application, where a calibration measure with an acceptable standard, such as the cuff, is typically taken at the doctor’s office. However, the frequent requirement of this event might grow the complexity of the use case. In comparison with previous results in [16], no restriction of timespan between calibration and estimation is imposed in post-processing. However, the dataset only includes relatively short recordings, with an average of 12 min per patient. This only allows to evaluate relatively small timespans. A long-term analysis should be conducted to quantify the stability of the performance with longer calibration delays.

As previously mentioned, the data used in this study comes from a controlled and restricted environment. ML methods are built based on the data available during the training of the model. Inevitably, this leads to the important question of generalization capability. This term describes the model’s ability to properly adapt to previously unseen data. A deeper study should be conducted to confirm the generalization capability, the robustness and potential applicability of the proposed ML methods and feature set to other related contexts. One limitation of this study is the small amount of data available. The model should be tested on different datasets, with typically larger sample size and different target populations. The PPG signals were recorded using a finger-clip sensor in transmission mode and consequently signals of good quality are obtained. It would be interesting to further investigate such an approach on signals collected at various measurement sites, such as wrist, upper arm or earlobe, and therefore to vary the sensors between transmission or reflection modes.

## Conclusion

Our study confirmed that features derived from the PPG morphology can be combined with ML methods to accurately track BP variations generated during anesthesia induction. The feature relevance analysis showed the importance of adding a calibration PPG-BP measure and highlighted the key role of features derived from the PPG 1^st^, 2^nd^ and 3^rd^ derivatives. The evaluation carried out showed a good agreement of the estimate with the invasive reference, as well as the model’s ability to track acute BP changes induced by anesthesia. Future work will investigate BP variations resulting from different physiological mechanisms. It will also determine how well the model adapts to other use cases of interest, such as the ambulatory monitoring of people with hypertension. Overall, the results highlighted the potential of ML models based on PPG-PWA features to help overcoming the current limitations of non-invasive BP monitoring–an important step towards more effective detection and management of hypertension in the future.
